# Sodium renders endothelial cells sticky for red blood cells

**DOI:** 10.3389/fphys.2015.00188

**Published:** 2015-06-30

**Authors:** Hans Oberleithner, Mike Wälte, Kristina Kusche-Vihrog

**Affiliations:** Medical Faculty, Institute of Physiology II, University of MünsterMünster, Germany

**Keywords:** endothelial glycocalyx, spironolactone, aldosterone, thrombosis, atomic force microscopy

## Abstract

Negative charges in the glycocalyx of red blood cells (RBC) and vascular endothelial cells (EC) facilitate frictionless blood flow through blood vessels. Na^+^ selectively shields these charges controlling surface electronegativity. The question was addressed whether the ambient Na^+^ concentration controls RBC-EC interaction. Using atomic force microscopy (AFM) adhesion forces between RBC and endothelial glycocalyx were quantified. A single RBC, mounted on an AFM cantilever, was brought in physical contact with the endothelial surface and then pulled off. Adhesion forces were quantified (i) after enzymatic removal of negative charges in the glycocalyx, (ii) under different ambient Na^+^ and (iii) after applying the intracellular aldosterone receptor antagonist spironolactone. Removal of negative surface charges increases RBC-EC interaction forces. A stepwise increase of ambient Na^+^ from 133 to 140 mM does not affect them. However, beyond 140 mM Na^+^ adhesion forces increase sharply (10% increase of adhesion force per 1 mM increase of Na^+^). Spironolactone prevents this response. It is concluded that negative charges reduce adhesion between RBC and EC. Ambient Na^+^ concentration determines the availability of free negative charges. Na^+^ concentrations in the low physiological range (below 140 mM) allow sufficient amounts of vacant negative charges so that adhesion of RBC to the endothelial surface is small. In contrast, Na^+^ in the high physiological range (beyond 140 mM) saturates the remaining negative surface charges thus increasing adhesion. Aldosterone receptor blockade by spironolactone prevents Na^+^ induced RBC adhesion to the endothelial glycocalyx. Extrapolation of *in vitro* experiments to *in vivo* conditions leads to the hypothesis that high sodium intake is likely to increase the incidence of thrombotic events.

## Introduction

Van der Waals forces comprise the attractive and repulsive forces between molecules other than those due to covalent bonds. They include the electrostatic interaction of ions with charged molecules. In the vascular system, such interactions occur selectively between plasma Na^+^ and the negatively charged glycocalyx (GC) of red blood cells (RBC) and vascular endothelial cells (EC). The properties of the endothelial glycocalyx determine tissue blood flow, capillary permeability and participate in the control of blood pressure (Weinbaum et al., 2007; Noble et al., 2008; Peters et al., 2012). In the presence of aldosterone (a steroid hormone usually present in blood under physiological conditions) high plasma Na^+^ stiffens endothelial cells accompanied by endothelial dysfunction (Oberleithner et al., 2007; Lang, 2011). Furthermore, high Na^+^ destroys the endothelial GC when chronically applied (Oberleithner et al., 2011). Taken together, high plasma Na^+^ either neutralizes the negative charges of the RBC and EC glycocalyx or even leads to a loss of the negative charges. The consequences are the same, namely the repulsive forces between RBC and EC will be attenuated. Since the physiology of blood flow in the vascular system strongly depends on the availability of sufficient amounts of negative surface charges (Vink et al., 1995) we quantified the forces between RBC and EC in different ambient Na^+^ concentrations. The tool of choice for adhesion measurements on individual living cells was an atomic force microscope used as a nanosensor (Radmacher et al., 1994). The focus was put on the quantification of forces that occur between RBC and EC in relation to Na^+^ homeostasis.

## Materials and methods

### Cell culture and RBC collection

The endothelial cell line EA.hy926 (kindly provided by Cora-Jean S. Edgell, University of North Carolina, Chapel Hill, NC, USA) was cultured in Dulbecco's modified Eagle's medium supplemented with 10% fetal calf serum and 10,000 U/ml penicillin/streptomycin as described elsewhere (Edgell et al., 1990). For experiments, cells were seeded on glass bottom Petri dishes and used after reaching confluence (48–72 h). Red blood cells were freshly collected from capillary blood of healthy volunteers (puncture of the finger tip). The blood was diluted 1:500 by adding HEPES-buffered saline to create an appropriate RBC suspension. No anti-coagulants were used.

### Solutions and reagents

Experiments were performed in HEPES (*N*-2-hydroxyethylpiperazine-*N'*-2-ethanesulfonic acid) buffered solution (140 mM NaCl, 5 mM KCl, 1 mM MgCl_2_, 1 mM CaCl_2_, 5 mM glucose, 10 mM HEPES; pH 7.4) supplemented with 1% fetal calf serum for maintaining glycocalyx integrity (Reitsma et al., 2007). Chemicals were purchased from Sigma-Aldrich (Munich, Germany) unless otherwise specified. Enzymatic digestion of the heparan sulfate residues in both RBC and EC were induced by a 30 min treatment with 1 mU/ml heparinase I (at 37°C) as described previously (Oberleithner et al., 2011). Experimental variations in ambient NaCl concentrations (from 133 to 148 mM) were osmotically balanced by mannitol added to the various solutions (30, 22, 16, 10, and 0 mM mannitol added to the respective solutions containing 133, 137, 140, 143, and 148 mM Na^+^). Aldosterone (d-aldosterone) and spironolactone (ICN Biochemicals, Irvine, USA) were used at final concentrations of 1.0 nM and 100 nM, respectively.

### Atomic force microscopy and adhesion measurements

**Figure 1:**
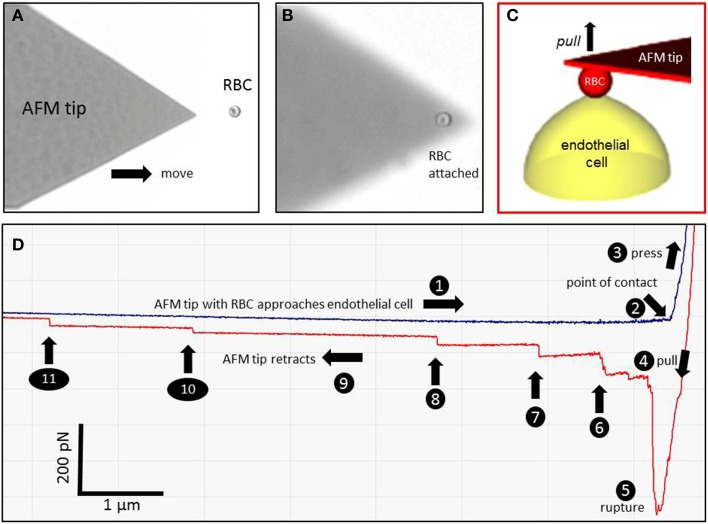
**Atomic force microscopy (AFM) cell adhesion measurements**. A red blood cell (RBC) is attached to the AFM tip **(A,B)** and brought in contact with an endothelial cell (EC) **(C)**. A representative force-distance curve is shown in **(D)**. The different events are numbered (1–11). (1) The RBC-functionalized AFM cantilever approaches the endothelial surface. (2) The RBC engages with the EC. (3) The RBC is pressed against the EC. (4) The RBC is pulled off the cell surface. (5) The RBC-EC interaction breaks down (“rupture”). (6–11) Single remaining RBC-EC bonds (“mini-ruptures”) break. They originate from plasma membrane tethers that are formed during AFM cantilever retraction.

AFM experiments were performed by using a CellHesion 200 instrument (JPK, Berlin, Germany) equipped with a Petri dish heater for maintaining 37°C. All experiments were analyzed using JPK Data Processing (software version 4.2.50). Arrow TL-1 tipless cantilevers (NanoAndMore GmbH, Wetzlar, Germany) were incubated prior to all experiments for 30 min in a solution of 1 mg/ml wheat germ agglutinin (WGA; Sigma Aldrich L 9640; dissolved in water). WGA makes the AFM cantilever sticky for RBC since it binds to N-acetyl-D-glucosamine and sialic acid known to be present in the RBC glycocalyx. Crucial experimental steps are documented in Figure 1. By means of a (blue) Eppendorf pipette tip a scratch (about 2 mm in width) in the endothelial cell layer was made in order to create a cell-free space. Then, the dish was washed (HEPES buffered saline) and the RBC suspension (2 μl) was pipetted into this free space. As a next step, the front part (apex) of the WGA-coated cantilever was brought in contact with a single RBC for 5–10 s using a maximal loading force of 1 nN in order to attach the RBC firmly to the AFM cantilever (Figures 1A–C). During the whole procedure the endothelial cell layer was always covered by HEPES buffered saline. Force-distance curves were obtained by probing EAhy629 cells with the RBC-carrying cantilever using a maximal loading force of 1 nN (Figure 1D). Further specific AFM parameters were as follows: average AFM cantilever spring constant: 0.02–0.025 N/m; average deflection sensitivity: 90 nm/V; z-length (pull-off distance): 50 μm; cantilever velocity during approach/retraction: 5 μm/s; cantilever contact time (RBC contact time with the endothelial cell): 1 s. In average, two force-distance curves were performed per one individual cell. Only force curves were evaluated that met a specific criterion as follows: The retraction curve after the initial spike must return by at least 50% of the spike's height toward the baseline within less than 1 μm of the retraction distance (which translates to less than 200 ms of retraction time). About 50% of the obtained force curves met this criterion. Positioning of the RBC-mounted cantilever on top of an individual endothelial cell was performed manually under visual control using the x,y drives of the instrument. Numbers of evaluated force curves are given in the respective figure legends.

## Statistical analysis

Box plots show IQR, median (band) and mean (square) values. Statistical analysis was done with STATISTICA 9 (StatSoft, Tulsa, USA). The normal distribution of data was checked using Shapiro-Wilk's W test. Statistical evaluations were done using Student's *t*-test. For the comparison of multiple groups, ANOVA followed by *post-hoc*-tests was used. Significance level was assumed as *p* ≤ 0.05.

## Results

**Figure 2:**
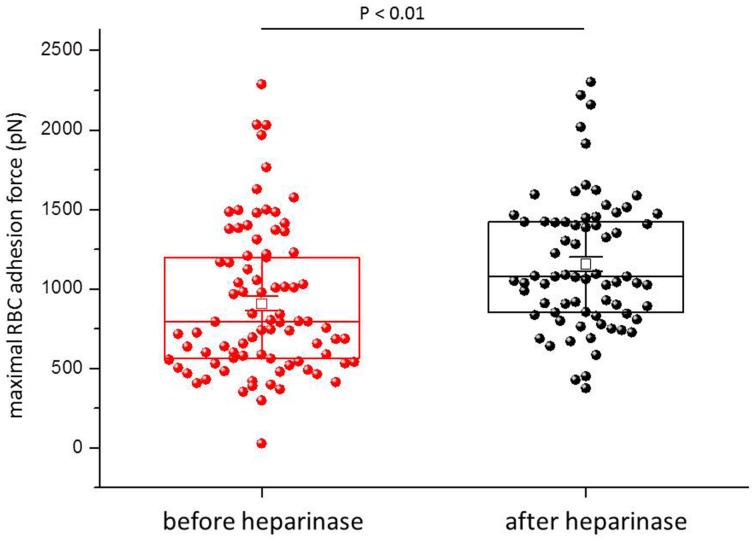
**Maximal RBC-EC interaction forces before and after heparinase treatment**. Each symbol represents the adhesion force of an endothelial cell.

**Figure 3:**
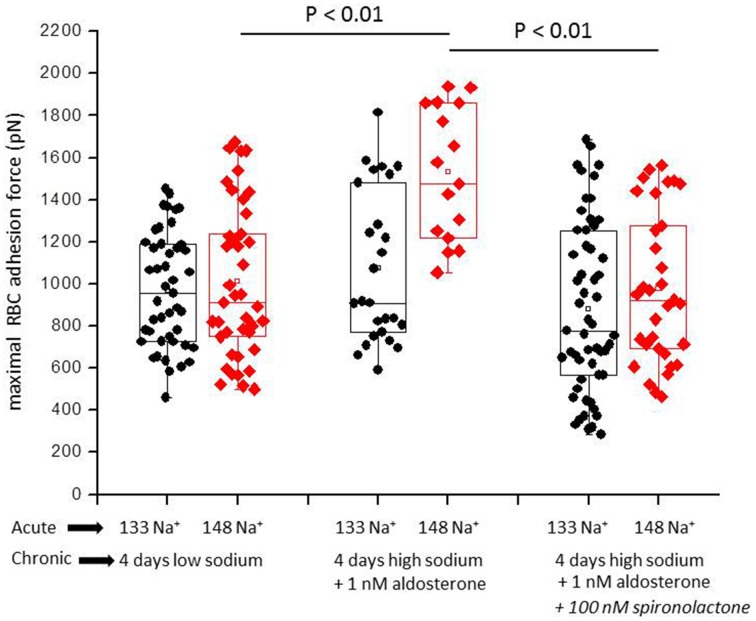
**Maximal RBC-EC interaction forces in different ambient Na^+^ concentrations**. “Acute” indicates measurements performed at a defined ambient Na^+^ concentration within a time window of 30 min. “Chronic” indicates that the cells under study were maintained for 4 days in either low Na^+^ (133 mM Na^+^) or high Na^+^ (148 mM Na^+^) culture medium prior to the acute experiments. Each symbol represents the adhesion force of an endothelial cell. Presence of aldosterone and spironolactone as indicated.

**Figure 4:**
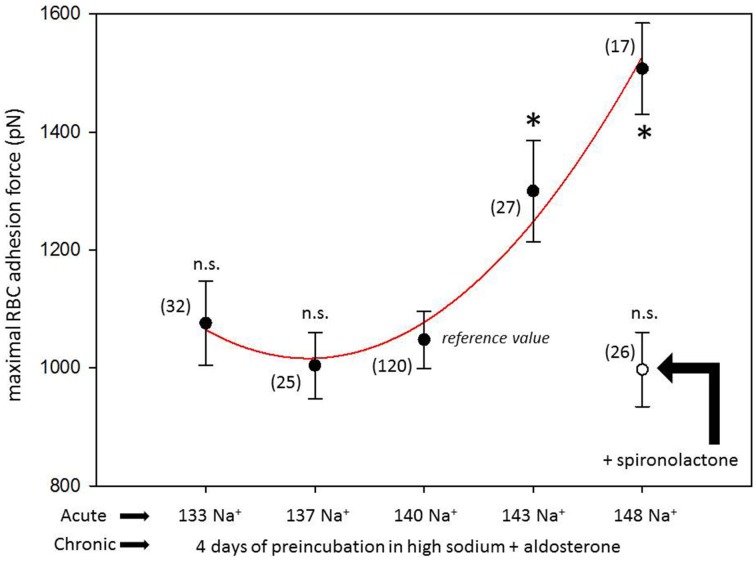
**Maximal RBC-EC interaction forces in different ambient Na^+^concentrations**. “Acute” indicates measurements performed at a defined ambient Na^+^ concentration within a time window of 30 min. “Chronic” indicates that all cells under study were maintained for 4 days in high Na^+^ (148 mM Na^+^ + 1 nM aldosterone) culture medium prior to the acute experiments. Presence of spironolactone as indicated. Symbols are mean values (±SE) of adhesion forces of individual cells (*n* = number of measurements given in parenthesis). The symbol ^*^ indicates that the mean value is significantly different (*P* < 0.05) in comparison to the 140 mM Na^+^ reference value. The symbol ^n.s.^ indicates that the mean value is not significantly different in comparison to the 140 mM Na^+^ reference value (*P* > 0.05).

**Figure 5:**
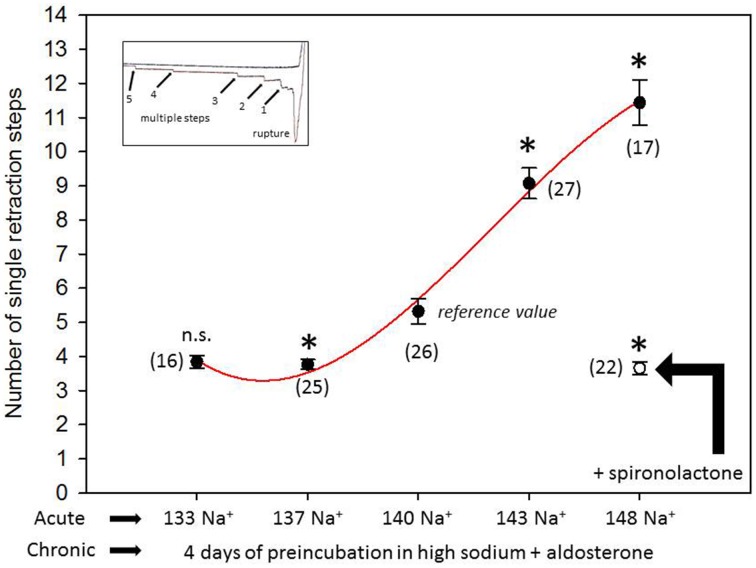
**Number of single retraction steps (rupture of the “mini-bonds” between RBC and EC) following the initial large peak (maximal adhesion force)**. “Acute” indicates measurements performed at a defined ambient Na^+^ concentration within a time window of 30 min. “Chronic” indicates that the cells under study were maintained for 4 days in high Na^+^ (148 mM Na^+^ + 1 nM aldosterone) culture medium prior to the acute experiments. Presence of spironolactone as indicated. Symbols are mean values (±SE) of adhesion forces of individual cells (*n* = number of measurements given in parenthesis). The symbol ^*^ indicates that the mean value is significantly different (*P* < 0.05) in comparison to the 140 mM Na^+^ reference value. The symbol ^n.s.^ indicates that the mean value is not significantly different in comparison to the 140 mM Na^+^ reference value (*P* > 0.05).

Figure 1 shows a representative force-distance curve. For analyses, the emphasis was put on the initial spike of the retraction curve (No. 5 in Figure 1D). This initial spike induced by the “rupture” of the bonds between RBC and endothelial cell surface was quantified (maximal RBC adhesion force). The single small steps following the initial spike (No. 6–11 in Figure 1D) are most probably related to membrane tethers that are being formed and then subsequently rupture during the retraction phase (Friedrichs et al., 2013). Net adhesion force between RBC and EC depends on the sum of attractive/repulsive forces. When an erythrocyte is brought in physical contact with the surface of an endothelial cell for a defined period of time (1 s), some force is necessary to separate these two surfaces from each other. As shown in Figure 2 a significantly larger force for separating RBC from the endothelial cell surface is necessary after polymeric heparan sulfate residues of the GC have been degraded by heparinase treatment. This is consistent with the view that after enzymatic removal of (some) negative charges located in the endothelial glycocalyx the attractive forces dominate, i.e., RBC-EC adhesion forces are pronounced. Figure 3 shows the dependence of the adhesion forces on ambient Na^+^ and the presence/absence of aldosterone. When cells were incubated for 4 days in low Na^+^ culture medium in absence of aldosterone and then acutely exposed to high Na^+^ RBC-EC interaction forces do not change. However, when cells were incubated for 4 days in high Na^+^ culture medium in presence of aldosterone and then acutely exposed to high Na^+^, adhesion forces increase. When spironolactone is added in a 100-fold concentration (related to the aldosterone concentration) to the 4-days culture, then RBC-EC interaction is insensitive to high Na^+^. In supplemental experiments in cells cultured in high Na^+^ (and in the presence of aldosterone) we acutely increased Na^+^ in small concentration steps, starting at 133 mM Na^+^ and finishing at 148 mM Na^+^ (Figure 4). RBC-EC interaction remains unaffected between 133 and 140 mM Na^+^ but sharply increases at higher Na^+^ concentrations. When cells were pretreated with spironolactone, RBC-EC interaction forces remain insensitive to ambient high Na^+^ consistent with the data of Figure 3. Figure 5 documents the number of single retraction steps following the initial rupture peak. These steps indicate “mini-ruptures” possibly due to individual membrane tethers that are formed when the RBC-functionalized AFM cantilever is retracted (Friedrichs et al., 2013). Significantly more “mini-ruptures” (steps) occur in ambient high Na^+^ indicating that RBC-EC interaction is elevated.

## Discussion

Plasma Na^+^ is known to influence vascular function. *In vitro*, any increase beyond 140 mM Na^+^ stiffens endothelial cells accompanied by a decrease of nitric oxide release (Oberleithner et al., 2007). *In vivo*, plasma Na^+^ in the high physiological range is accompanied by elevated blood pressure (He et al., 2005; Suckling et al., 2013; Kliche et al., 2015), an observation that supports the *in vitro* results (Oberleithner et al., 2007). Since sustained elevated blood pressure affects the morphology of blood vessels, it is assumed to also affect RBC-EC adhesion. In cultured endothelium chronic high Na^+^ exposure damages the endothelial GC (Oberleithner et al., 2011). Under these conditions, the negatively charged heparan sulfate residues of the GC have been shed which renders the endothelial surface vulnerable. A damaged GC can be more easily penetrated by RBC as proposed recently (Lee et al., 2014). This scenario is thought to have also functional consequences for RBC. It was shown *in vitro* that a damaged endothelial GC leaves fingerprints on the RBC surface, i.e., RBC glycocalyx is also damaged when the endothelial GC is impaired (Oberleithner, 2013). These *in vitro* results are supported by clinical observations that endothelial dysfunction is often combined with impaired RBC function (Hebbel et al., 1981; Bonomini et al., 2002; Vaziri, 2004; Vogt et al., 2004; Pot et al., 2011; Serroukh et al., 2012; Varez-Llamas et al., 2012; Vlahu et al., 2012). Based on the arguments that the physical interaction of RBC with the endothelial surface of blood vessels depends on the functional state of negatively charged GC and that plasma Na^+^ is a major determinant for GC electronegativity, it led us to measure the interaction forces between RBC and endothelium in quantitative terms by using AFM. Analyses of the force-distance curves indicate significant adhesion between RBC and EC. Maximal adhesion as analyzed in the present study is defined as the force necessary to disrupt the bonds between RBC and EC. As shown here, maximal adhesion is reduced when the endothelial GC is intact and increases when negatively charged proteoglycan associated residues (heparan sulfate) are removed.

Adhesion forces between RBC and EC can be explained in different ways but none of them will tell us the “true” (complete) story. Adhesion can be influenced by specific bonds, van der Waal's interaction, electrostatic attraction/repulsion but also by non-specific forces. One of them is called “depletion interaction.” Depletion interaction is the result of a lower macromolecule concentration near the cell surface as compared with the bulk medium (Yang et al., 2010). This exclusion of macromolecules near the cell surface leads to an osmotic gradient. Relating this process to RBC-EC interaction, albumin (which is present in the solution) is displaced from the depletion zone into the bulk phase leading to an attractive force. Such a scenario could explain why we observe significant adhesion (attraction forces) between RBC and EC despite the repulsive forces generated by the glycocalyx of the two cell types.

High Na^+^ stiffens endothelial cells (Oberleithner et al., 2007) and renders them sticky (present study). The “stiff endothelial cell syndrome” (Lang, 2011) appears related to amiloride sensitive epithelial Na^+^ channels (EnNaC) but also to the break-down of the endothelial GC barrier (Warnock et al., 2014). High Na^+^ triggers EnNaC function (Korte et al., 2014) and, applied chronically, is known to damage the GC (Oberleithner et al., 2011). Up to this point, it is not clear whether the enhanced interaction between RBC and EC is caused by Na^+^ induced EnNaC activation and/or mainly by Na^+^ induced GC shedding. Definitely, the GC is involved since enzymatic removal of heparan sulfate residues increases adhesion. It is known that all three epithelial sodium channel subunits possess numerous Asn (*N*)-linked glycosylation sites at their large ectodomains (Canessa et al., 1994; Rotin et al., 2001) and it is tempting to speculate that whenever this plasma membrane sodium channel is increasingly expressed in endothelial cells—e.g., at high Na^+^ conditions—the endothelial surface layer, i.e., the GC, is impaired. This would explain why the “stiff endothelial cell syndrome” is not only characterized by EnNaC upregulation, cell stiffening and endothelial dysfunction but also by an altered GC (Drüppel et al., 2013; Wiesinger et al., 2013; Paar et al., 2014; Padberg et al., 2014).

It is worth analyzing the force—distance curves in more detail. When the RBC is brought in contact with the endothelial cell surface for a time period of 1 s, a significant retraction force is necessary to detach the RBC from the EC surface. This finding supports the view that, by using sufficient force (i.e., vertical pressure onto the endothelial surface), RBC can “penetrate” the electrically repulsive GC (Lee et al., 2014) and, if this happens, become engaged with yet unidentified structures of the plasma membrane. A potential candidate for such a structure could be the large extracellular domains of EnNaC (Snyder et al., 1994). This idea is based on the observation that EnNaC down-regulation by spironolactone (Drüppel et al., 2013) and thus reduced EnNaC abundance in the endothelial plasma membrane dampens adhesion.

The stepwise decrease of the adhesion forces as identified in the force-distance curves (Figure 1D) indicates multiple interaction sites between RBC and EC that “break” upon retraction (“mini-ruptures”). Such a pattern is known to be caused by the formation of plasma membrane tubes when two cells are being forced to detach from each other after close physical contact (Friedrichs et al., 2013). High Na^+^ increases the number of such retraction steps (“mini-ruptures”). This finding is consistent with the view that high Na^+^ weakens the (electronegative) repulsive forces resulting in increased adhesion.

Clinical relevance can be derived from the observation that spironolactone reduces RBC-EC interaction forces. This drug is known to reduce endothelial cell stiffness *in vitro* (Drüppel et al., 2013; Paar et al., 2014) but also improves cardiovascular function *in vivo* (Pitt et al., 1999). It can be concluded that spironolactone antagonizes aldosterone action not only in kidney but also in other organs and tissues including the vascular system (Funder, 2013, 2015). Extrapolation of *in vitro* observations to *in vivo* conditions leads to the assumption that high Na^+^ could facilitate harmful RBC-EC interactions in humans. The latter could happen in arteries at sites where the GC is thinned and thus RBC interaction with the vessel wall is likely to occur. Plasma Na^+^ concentration is known to increase after a salty meal for a few millimoles per liter blood (He et al., 2005; Suckling et al., 2013) and is also expected to increase over night (water loss due to “perspiratio insensibilis”). This scenario could explain why acute vascular problems (thrombotic events, cardiac infarction) often occur in early morning.

### Limitations of the study

We are aware of several limitations of the present study. Here we mention at least three of them. (i) Observations are based on cultured cells only. We do not yet know whether and to what extent these *in vitro* findings can be extrapolated to *in vivo* conditions. (ii) Experiments were performed in one particular endothelial cell type only. It is likely that RBC-EC interaction could be different in various types of blood vessels. (iii) The present experimental design ignores the potential influence of hemodynamics (e.g., streaming fluid, shear stress, etc).

Nevertheless, the *in vitro* results of the present study may open new perspectives on the influence of plasma sodium concentration in the interaction between red blood cells and vascular endothelium.

### Conflict of interest statement

The authors declare that the research was conducted in the absence of any commercial or financial relationships that could be construed as a potential conflict of interest.
